# Supplementary material for: Prediction of occult tumor progression via platelet RNAs in a mouse melanoma model: a potential new platform for early detection of cancer
Source: J Transl Med. 2022 Feb 5;20:71. doi: 10.1186/s12967-022-03268-z (PMC8817485; doi:10.1186/s12967-022-03268-z)
Supplement: Supplementary file 1 — Additional file 1: Fig. S1. Platelet RNA profiles of mice inoculated with B16F10 cells are consistent with previous studies. a, Images of mice after terminal blood collection. Representative images of mice in O group (optimal inoculation group, mice inoculated with 1 × 10^5^ B16F10 cells) (left) and S group (suboptimal inoculation group, mice inoculated with 2 × 10^3^ B16F10 cells) with HE-stained histological images of tissue from inoculation site (right). b, Platelet mRNA sequencing data of known platelet-abundant genes with a dashed line showing the average read count of our data. c, Pearson’s correlation (color bar) matrix of our mRNA sequencing data of platelets and PBMCs (columns and rows). S: platelet suboptimal inoculation group; O: platelet optimal inoculation group; C: platelet negative control group. PS: PBMC suboptimal inoculation group; PO: PBMC optimal inoculation group; PC: PBMC negative control group. d, Heatmap of previously reported differentially expressed genes between platelets and PBMCs from our sequencing data. Fig. S2. PBMC RNA profiles of mice inoculated with an optimal or suboptimal number of B16F10 cells. a, Correlation plots of mRNAs detected in PBMCs of suboptimal inoculation group (S group, mice inoculated with 2 × 10^3^ B16F10 cells), negative control group (C group, mice injected with HBSS) and optimal inoculation group (O group, mice inoculated with 1 × 10^5^ B16F10 cells) mice, including highlighted increased (red) and decreased (blue) PBMC mRNAs. NRC, normalized read counts (mean of group). r value calculated from Pearson's correlation test. b, Venn diagram of differentially expressed genes from pairwise comparisons. c, Heatmap of hierarchical clustering of PBMC mRNA profiles of S group (beige), C group (green) and O group (orange). Data pooled from (a, b) n = 5 biologically independent experiments with n = 24 (S group) or n = 25 (C group) and n = 20 (O group) mice or data representing all 5 independent experiments (c). d, Top GO terms of [file 12967_2022_3268_MOESM1_ESM.docx]

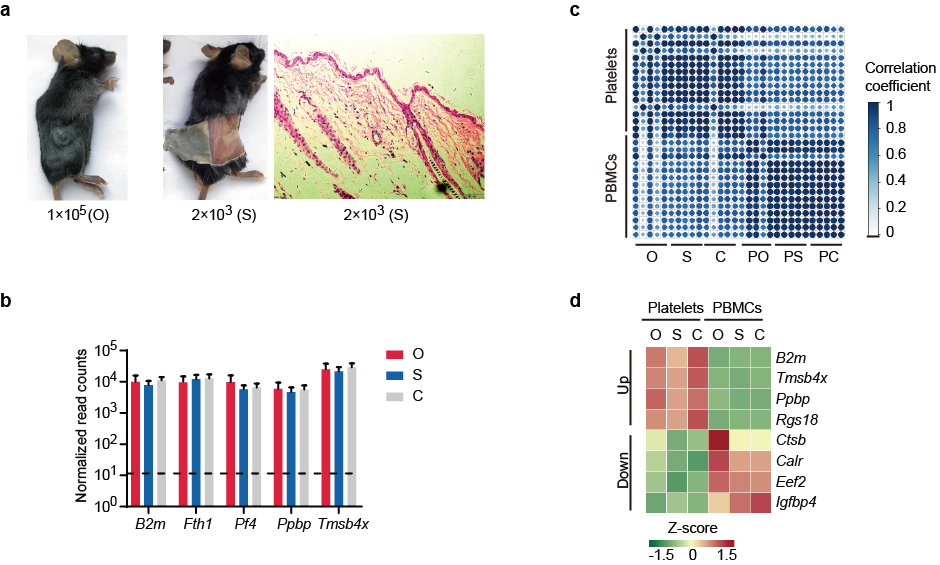
 Fig. S1 Platelet RNA profiles of mice inoculated with B16F10 cells are consistent with previous studies.

**a**, Images of mice after terminal blood collection. Representative images of mice in O group (optimal inoculation group, mice inoculated with 1 × 10^5^ B16F10 cells) (left) and S group (suboptimal inoculation group, mice inoculated with 2 × 10^3^ B16F10 cells) with [HE-stained histological images](https://diagnosticpathology.biomedcentral.com/articles/10.1186/s13000-021-01126-y) of tissue from inoculation site (right). **b**, Platelet mRNA sequencing data of known platelet-abundant genes with a dashed line showing the average read count of our data. **c**, Pearson’s correlation (color bar) matrix of our mRNA sequencing data of platelets and PBMCs (columns and rows). S: platelet suboptimal inoculation group; O: platelet optimal inoculation group; C: platelet negative control group. PS: PBMC suboptimal inoculation group; PO: PBMC optimal inoculation group; PC: PBMC negative control group. **d**, Heatmap of previously reported differentially expressed genes between platelets and PBMCs from our sequencing data.


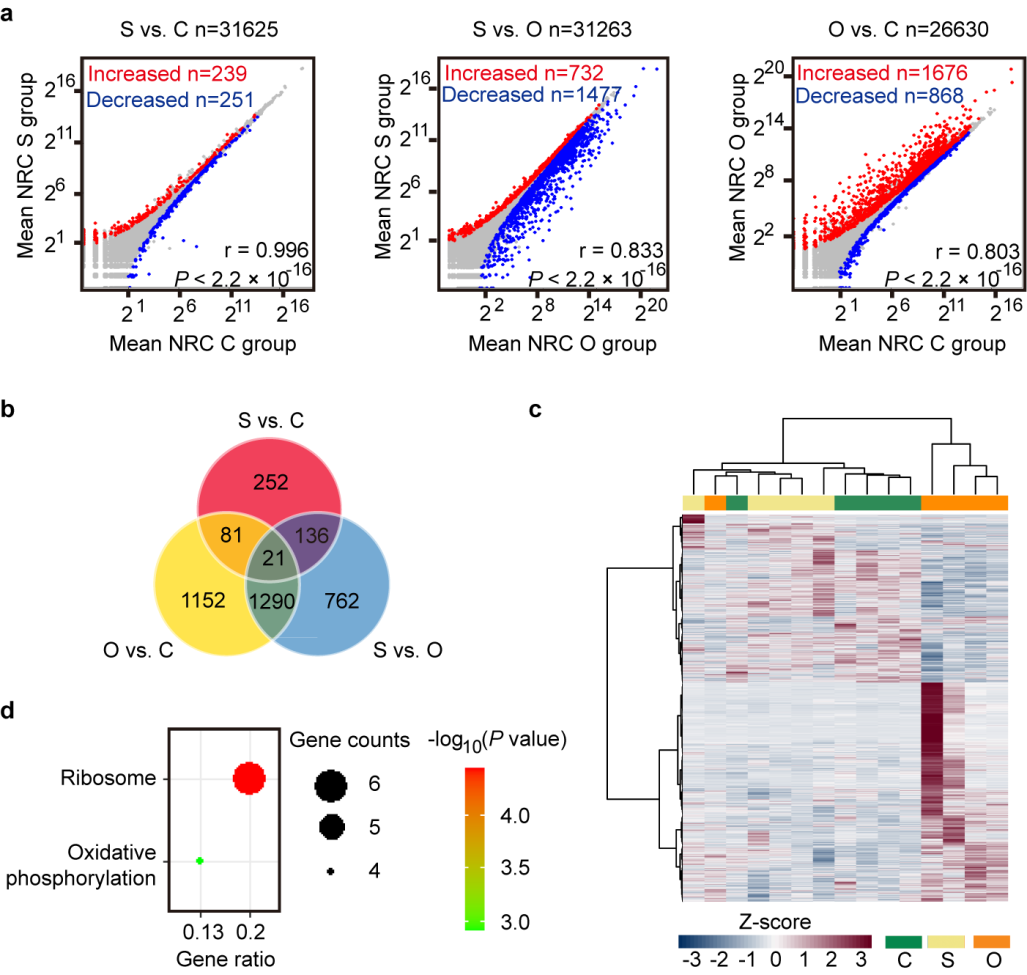


Fig. S2 PBMC RNA profiles of mice inoculated with an optimal or suboptimal number of B16F10 cells.

**a**, Correlation plots of mRNAs detected in PBMCs of suboptimal inoculation group (S group, mice inoculated with 2 × 10^3^ B16F10 cells), negative control group (C group, mice injected with HBSS) and optimal inoculation group (O group, mice inoculated with 1 × 10^5^ B16F10 cells) mice, including highlighted increased (red) and decreased (blue) PBMC mRNAs. NRC, normalized read counts (mean of group). r value calculated from Pearson's correlation test. **b**, Venn diagram of differentially expressed genes from pairwise comparisons. **c**, Heatmap of hierarchical clustering of PBMC mRNA profiles of S group (beige), C group (green) and O group (orange). Data pooled from (**a**, **b**) *n* = 5 biologically independent experiments with *n* = 24 (S group) or *n* = 25 (C group) and *n* = 20 (O group) mice or data representing all 5 independent experiments (**c**). **d**, Top GO terms of pathway enrichment analysis of eligible eDEGs (strategy same as Fig. 3a, details in “Methods”) in PBMCs with reference from KEGG pathways. Adjusted *P* value < 0.05, Benjamini and Hochberg method.


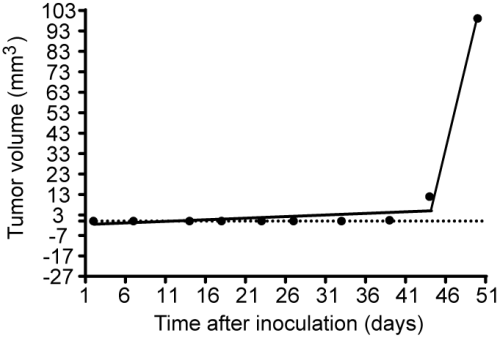


Fig. S3 An example of the selected models from Joinpoint multi-phase regression analyses.

Tumor growth data from one mouse in early-early tumor group (E group).


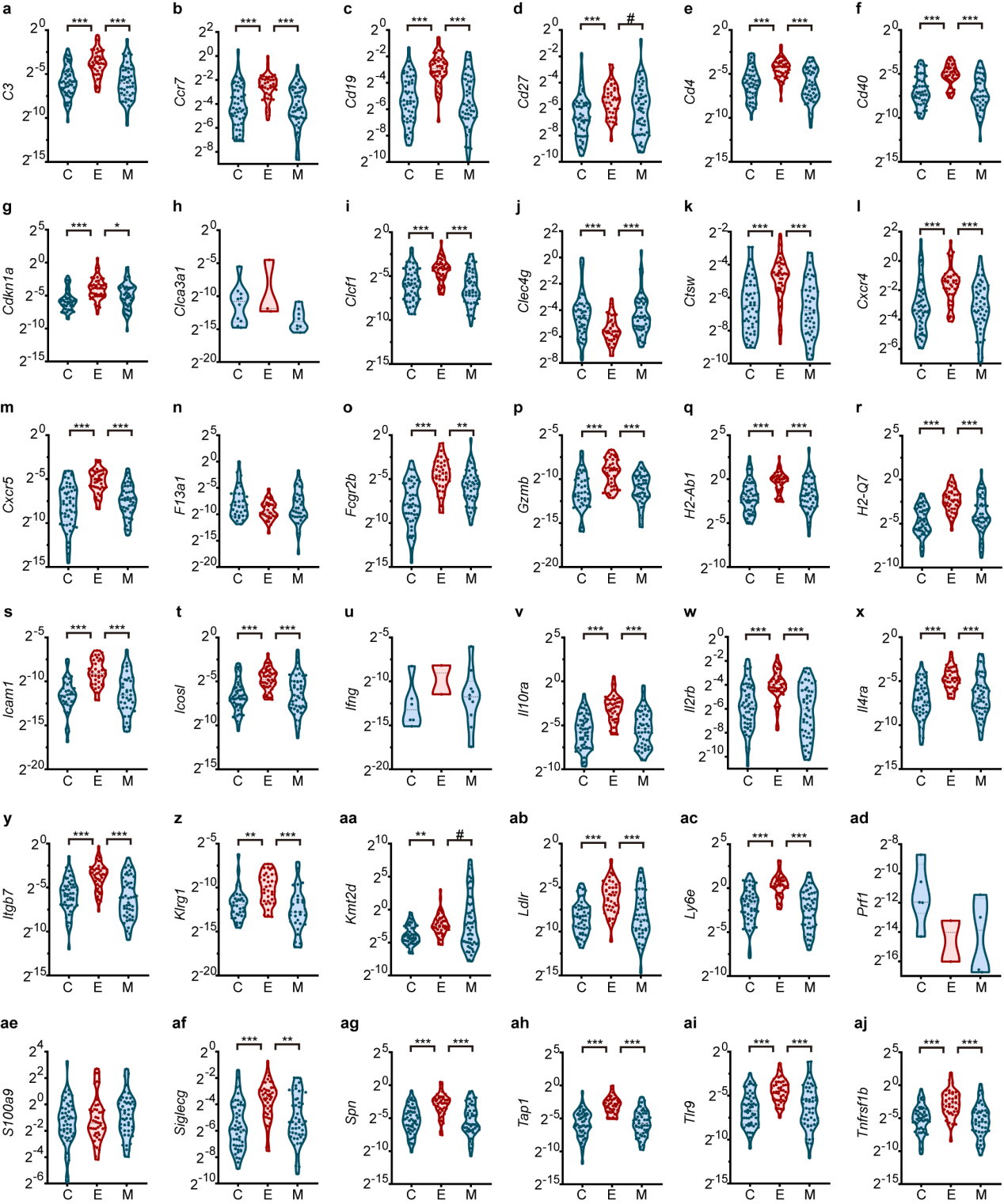


Fig. S4 qPCR verifications of the expression levels of selected 36 genes in the mouse cohort.

Violin plots of normalized gene expression levels (2^ΔCt(Ref-Gene)^) of 36 selected genes in three groups of the mouse cohort. C: negative control; E: early-early tumor; M: macroscopic melanoma. **P* < 0.05, ***P* < 0.01, ****P* < 0.001, Kruskall-Wallis test. Data without significance tags representing non-significant for analyses between three groups (**h**, **n**, **u**, **ad**, **ae**) (detailed statistics including *n* values and *P* values see Supplementary Table 5).


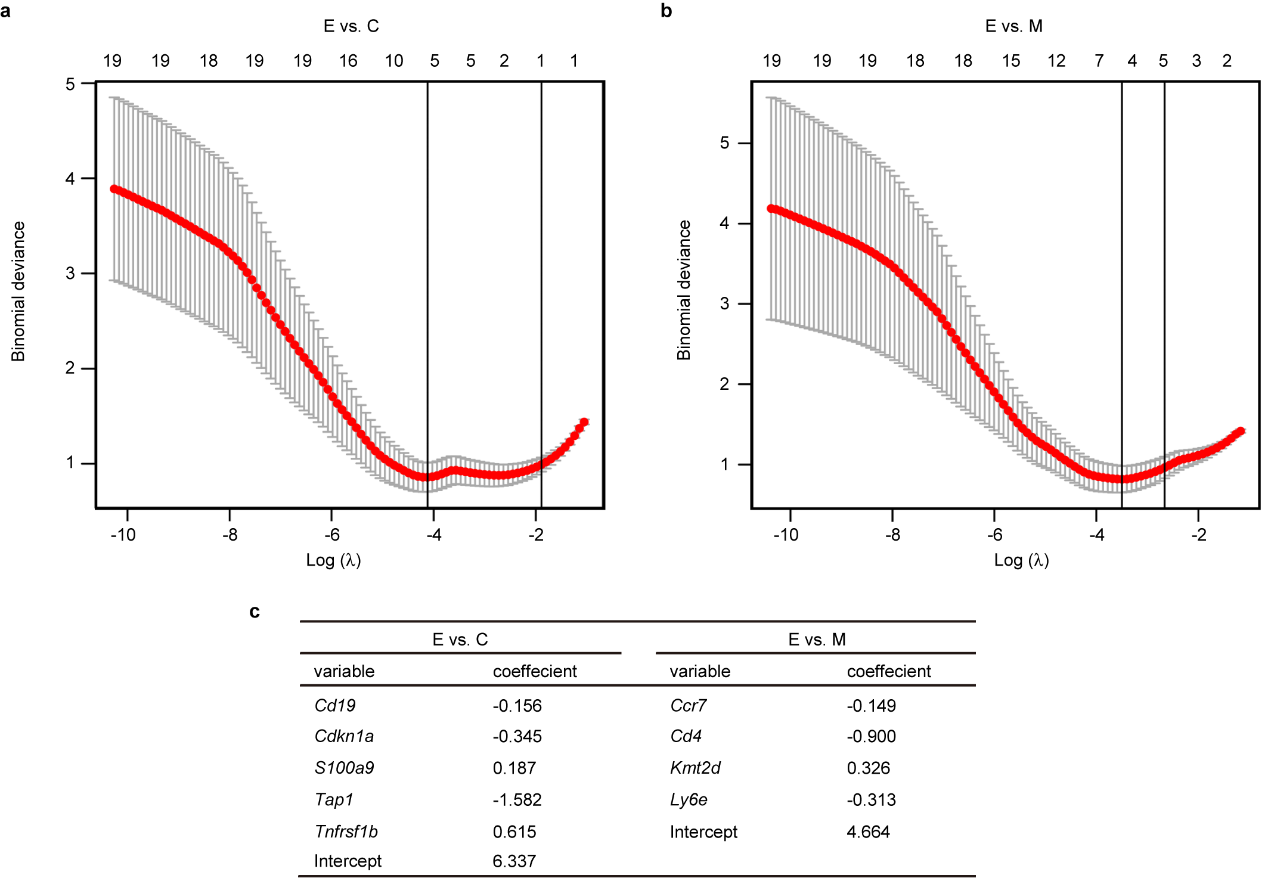


Fig. S5 LASSO regression model construction and variable selection for predicting occult tumor progression in mouse cohort.

**a**, **b**, Ten-fold cross-validation for the selection of the penalty term λ with the binomial deviance as measures of the predictive performance of the fitted models. The dependent variable groups: E vs. C (**a**) or E vs. M (**b**). **c**, Coefficients derived from LASSO regression. E, early-early tumor (occult tumor that progressed into macroscopic tumor later); C, negative control group; M, macroscopic melanoma group. Numbers of samples included in LASSO regression: E, *n* = 40; C, *n* = 50; M, *n* = 45. Numbers of variables included in LASSO regression: E vs. C, *n* = 29; E vs. M, *n* = 30. The prediction score formulas for the discrimination of E group from C group (Score_EC_) or from M group (Score_EM_) established as follows: Score_EC_ = 6.337 – 0.156 × (Ct*_Cd19_* – Ct_Ref_) – 0.345 × (Ct*_Cdkn1a_* – Ct_Ref_) + 0.187 × (Ct*_S100a9_* – Ct_Ref_) – 1.582 × (Ct*_Tap1_* – Ct_Ref_) + 0.615 × (Ct*_Tnfrsf1b_* – Ct_Ref_); Score_EM_ = 4.664 – 0.149 × (Ct*_Ccr7_* – Ct_Ref_) – 0.900 × (Ct*_Cd4_* – Ct_Ref_) + 0.326 × (Ct*_Kmt2d_* – Ct_Ref_) – 0.313 × (Ct*_Ly6e_* – Ct_Ref_).


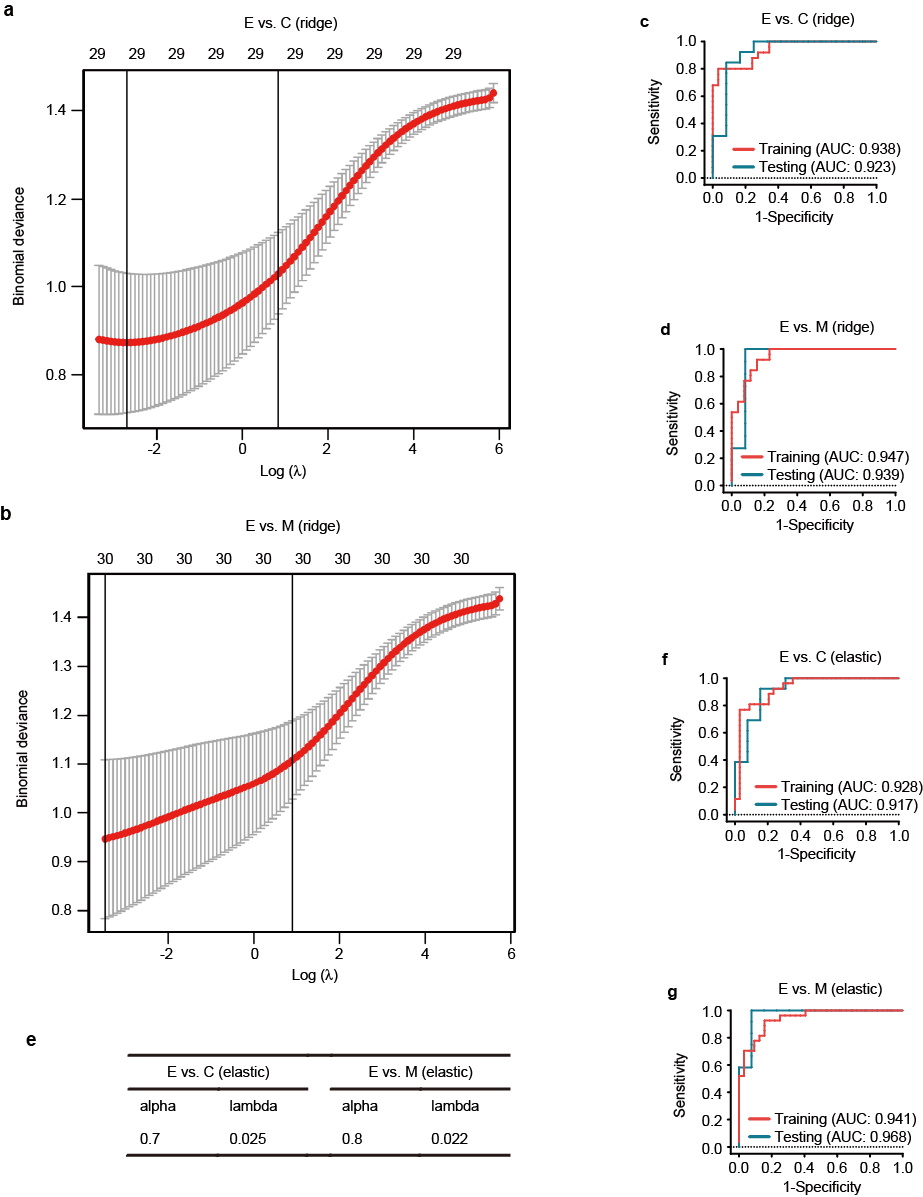


Fig. S6 Ridge and elastic net regression model construction and variable selection for predicting occult tumor progression in mouse cohort.

**a**, **b**, Ten-fold cross-validation for the selection of the penalty term λ with the binomial deviance as measures of the predictive performance of the fitted models for ridge regression (coefficients derived from LASSO regression see Supplementary Table 6). The dependent variable groups: E vs. C (**a**) or E vs. M (**b**). ROC curves for the diagnostic performances of the prediction score formulas generated from ridge **(c, d)** and elastic net **(f, g)** regression in the mouse cohort. ROC curves for the discrimination of early-early tumor (occult tumor, E group) from negative control group (C group) (**c, f**, E vs. C) or from macroscopic melanoma group (M group) (**d, g**, E vs. M). The prediction score formulas for the discrimination of E group from C group (Score_EC_) or from M group (Score_EM_) established as follows: Score = Intercept + Σ Coefficient × (Ct_Variable_ – Ct_Ref_). Probability statistics calculated according to the prediction score formulas generated from regression analyses: Probability = e^Score^ / (1 + e^Score^). 95% CI of AUC: training data 0.879-0.997, testing data 0.806-1.000 (**c**); training data 0.891-1.000, testing data 0.822-1.000 (**d**); training data 0.861-0.994, testing data 0.807-1.000 (**f**); training data 0.887-0.995, testing data 0.901-1.000 (**g**). **e**, Elastic net regression’s optimal alpha and lamda combination. E, early-early tumor (occult tumor that progressed into macroscopic tumor later); C, negative control group; M, macroscopic melanoma group. Numbers of samples included in regression: E, *n* = 40; C, *n* = 50; M, *n* = 45. Numbers of variables included in regression: E vs. C, *n* = 29; E vs. M, *n* = 30.

Table S1 Enriched KEGG pathways of differentially expressed mRNAs in platelets of suboptimal inoculation group.

| Term | Count | Gene ratio | *P* value | *P* adjust |
| --- | --- | --- | --- | --- |
| Cytokine-cytokine receptor interaction | 24 | 0.110 | 0.000 | 0.000 |
| Cell adhesion molecules | 20 | 0.091 | 0.000 | 0.000 |
| Epstein-Barr virus infection | 18 | 0.082 | 0.000 | 0.000 |
| Human T-cell leukemia virus 1 infection | 18 | 0.082 | 0.000 | 0.001 |
| Th1 and Th2 cell differentiation | 14 | 0.064 | 0.000 | 0.000 |
| Th17 cell differentiation | 14 | 0.064 | 0.000 | 0.000 |
| Phagosome | 14 | 0.064 | 0.000 | 0.002 |
| Human immunodeficiency virus 1 infection | 14 | 0.064 | 0.002 | 0.022 |
| Hematopoietic cell lineage | 12 | 0.055 | 0.000 | 0.000 |
| Staphylococcus aureus infection | 11 | 0.050 | 0.000 | 0.003 |
| Lysosome | 11 | 0.050 | 0.000 | 0.004 |
| Tuberculosis | 11 | 0.050 | 0.005 | 0.039 |
| Allograft rejection | 10 | 0.046 | 0.000 | 0.000 |
| Rheumatoid arthritis | 10 | 0.046 | 0.000 | 0.001 |
| Viral myocarditis | 10 | 0.046 | 0.000 | 0.001 |
| Antigen processing and presentation | 10 | 0.046 | 0.000 | 0.001 |
| Systemic lupus erythematosus | 10 | 0.046 | 0.004 | 0.030 |
| Primary immunodeficiency | 9 | 0.041 | 0.000 | 0.000 |
| Autoimmune thyroid disease | 9 | 0.041 | 0.000 | 0.002 |
| Viral protein interaction with cytokine and cytokine receptor | 9 | 0.041 | 0.001 | 0.006 |
| Chagas disease | 9 | 0.041 | 0.001 | 0.009 |
| Intestinal immune network for IgA production | 8 | 0.037 | 0.000 | 0.000 |
| Inflammatory bowel disease | 8 | 0.037 | 0.000 | 0.002 |
| Graft-versus-host disease | 8 | 0.037 | 0.000 | 0.002 |
| Type I diabetes mellitus | 8 | 0.037 | 0.000 | 0.004 |
| B cell receptor signaling pathway | 8 | 0.037 | 0.001 | 0.008 |
| PD-L1 expression and PD-1 checkpoint pathway in cancer | 8 | 0.037 | 0.001 | 0.013 |
| T cell receptor signaling pathway | 8 | 0.037 | 0.004 | 0.031 |
| Malaria | 6 | 0.027 | 0.003 | 0.023 |
| Glycosaminoglycan biosynthesis - chondroitin sulfate / dermatan sulfate | 5 | 0.023 | 0.000 | 0.002 |
| Asthma | 5 | 0.023 | 0.000 | 0.004 |

Table S2 Enriched KEGG pathways of differentially expressed mRNAs in PBMCs of suboptimal inoculation group.

| Description | Count | Gene ratio | *P* value | *P* adjust |
| --- | --- | --- | --- | --- |
| Ribosome | 6 | 0.200 | 0.000 | 0.002 |
| Oxidative phosphorylation | 4 | 0.133 | 0.001 | 0.044 |

Table S3 Joinpoint multi-phase regression statistics for all mice in early-early group of the mouse cohort.

| Mice | Joinpoint (days) | *P* value |
| --- | --- | --- |
| E1 | 34 | 0.024 |
| E2 | 29 | 0.036 |
| E3 | 34 | 0.012 |
| E4 | 34 | 0.019 |
| E5 | 34 | 0.045 |
| E6 | 28 | 0.003 |
| E7 | 23 | 0.000 |
| E8 | 23 | 0.007 |
| E9 | 38 | 0.000 |
| E10 | 19 | 0.007 |
| E11 | 28 | 0.000 |
| E12 | 28 | 0.000 |
| E13 | 44 | 0.000 |
| E14 | 33 | 0.000 |
| E15 | 27 | 0.000 |
| E16 | 27 | 0.000 |
| E17 | 27 | 0.000 |
| E18 | 33 | 0.000 |
| E19 | 27 | 0.000 |
| E20 | 27 | 0.000 |
| E21 | 23 | 0.000 |
| E22 | 27 | 0.000 |
| E23 | 21 | 0.003 |
| E24 | 21 | 0.000 |
| E25 | 31 | 0.000 |
| E26 | 23 | 0.003 |
| E27 | 23 | 0.029 |
| E28 | 23 | 0.000 |
| E29 | 29 | 0.000 |
| E30 | 23 | 0.003 |
| E31 | 23 | 0.003 |
| E32 | 35 | 0.000 |
| E33 | 25 | 0.000 |
| E34 | 25 | 0.003 |
| E35 | 20 | 0.000 |
| E36 | 25 | 0.011 |
| E37 | 25 | 0.005 |
| E38 | 25 | 0.000 |
| E39 | 20 | 0.000 |
| E40 | 25 | 0.000 |
| E41 | 25 | 0.004 |
| E42 | 20 | 0.018 |
| E43 | 25 | 0.015 |
| E44 | 25 | 0.015 |

*P* value, the statistic significance of the change in tumor growth trend over time calculated via a Monte Carlo Permutation method

Table S4 Primers for qPCR experiments.

| Ensembl Gene ID | Gene Symbol | Forward | Reverse |
| --- | --- | --- | --- |
| ENSMUSG00000029580 | *Actb** | TTCTTTGCAGCTCCTTCG | GACCAGCGCAGCGATATC |
| ENSMUSG00000057666 | *Gapdh** | CATCACCATCTTCCAGGA | TAGACTCCACGACATACTC |
| ENSMUSG00000025534 | *Gusb** | TATGAACGGGAAGCAATC | ATAATAATGGGCACTGTTGA |
| ENSMUSG00000027523 | *Gnas** | GCTTGCTTAGATGTTCCA | CTTTATTTCTGTTGCTGCTTT |
| ENSMUSG00000035242 | *Oaz1** | CAGAATAAACGCTCACTC | TTCAAAGGAGACTGCTAT |
| ENSMUSG00000024164 | *C3* | CATAGCCAAGTTCCTGTA | ATCTTCTTATCGCCATCC |
| ENSMUSG00000037944 | *Ccr7* | CCTGGTTGAGTAGTCTTC | TTGCTAAGTGTGGAGATAA |
| ENSMUSG00000030724 | *Cd19* | AGTATATGTCATCTTCTGTATGGT | GTCAGTCATTCGCTTCCT |
| ENSMUSG00000030336 | *Cd27* | GACTGACTGCCGTGAGAG | CAACTTACAGAGAAGACAGAACC |
| ENSMUSG00000023274 | *Cd4* | ACCTTGGATAGCAACTCTAA | TGGAACCACTGACAACTT |
| ENSMUSG00000017652 | *Cd40* | TTGGAGTTATGGAGATGG | ATGACTGATTGGAGAAGAA |
| ENSMUSG00000023067 | *Cdkn1a* | CTCCTTGTCACCTCTAAG | ATATTACGGTTGAGTCCTAA |
| ENSMUSG00000056025 | *Clca3a1* | TAGAAGATAGAATGGCACTAA | TTGAATGAGGAACTGTTAATC |
| ENSMUSG00000040663 | *Clcf1* | ACTACATATCCAGTTCAGG | CTCCAGCATAAACCCTAC |
| ENSMUSG00000074491 | *Clec4g* | GGTGGCTCCTATTCCTTGA | CAGGCTGAGTGGCTCTTAT |
| ENSMUSG00000024910 | *Ctsw* | ATATCTAACTGTCCTCAAC | ACCTTCTTGTACTTCTTG |
| ENSMUSG00000045382 | *Cxcr4* | GTAGAACTGTAGAGGAAGAA | CGGAATGAAGAGATTATGC |
| ENSMUSG00000047880 | *Cxcr5* | ATCTTCCTCCTGATTATG | TATTCCTCTGTGGTATAG |
| ENSMUSG00000039109 | *F13a1* | GAAGTTATGGAAGAGAAGAT | TAGATGCTAATGCTGAGA |
| ENSMUSG00000026656 | *Fcgr2b* | ACAGACACTACGAGATTG | ACGGCAGTAAGTTGATTA |
| ENSMUSG00000015437 | *Gzmb* | AAGAAGTAGAATGTTTGC | CTGTATGAAAGTTCTGTAG |
| ENSMUSG00000073421 | *H2-Ab1* | AATGTCTCTGCTCCGAAT | AGGTGGTGGATACAATAGT |
| ENSMUSG00000060550 | *H2-Q7* | AGAGTATGTGACCTTGATTGTTA | GGTTCTCCATCTGCCATT |
| ENSMUSG00000037405 | *Icam1* | ACTGGACTATAATCATTC | CCTTCTGTAACTTGTATA |
| ENSMUSG00000000732 | *Icosl* | AACTTGAGTGGTCTGTATG | GACTTGTAAGGCAGGTAG |
| ENSMUSG00000055170 | *Ifng* | TTAACTCAAGTGGCATAG | TGATTCAATGACGCTTAT |
| ENSMUSG00000032089 | *Il10ra* | GAATTAACAAGGCAGAGT | ACAGAATAGCAGCATAGA |
| ENSMUSG00000068227 | *Il2rb* | ACTACATTGAACCATACTTG | CTGCTTGAGGCTTAATAC |
| ENSMUSG00000030748 | *Il4ra* | TACTGGTATTCTGCCTTAG | AGACTGTTCTGAGATGTG |
| ENSMUSG00000001281 | *Itgb7* | GGTGGATTCATCAACTGT | ATGTAATCTTGGTATCTAACTCA |
| ENSMUSG00000030114 | *Klrg1* | TAAGAAGGTCCTATACTG | CTAATGGCATATCATAACA |
| ENSMUSG00000048154 | *Kmt2d* | CAACAACAGCAACAACAAC | ACCATGTGACATCATTCCT |
| ENSMUSG00000032193 | *Ldlr* | TTCTCCCATCTTCATTTCCT | TCCTCCGTGTTAGTGTTG |
| ENSMUSG00000022587 | *Ly6e* | AAGACCATTACTGTATCA | GAGATTGAGATTGACATT |
| ENSMUSG00000037202 | *Prf1* | TTAATAGCGACACAGTAGAGT | TGAAGTCAAGGTGGAGTG |
| ENSMUSG00000056071 | *S100a9* | TCAGACAAATGGTGGAAG | GCATCATACACTCCTCAA |
| ENSMUSG00000030468 | *Siglecg* | GACACCTATTCTGATTAC | CCTTAACTACTTTACCATT |
| ENSMUSG00000051457 | *Spn* | GCAGCATCTACATCTATCTCTA | GTCCACTGGTCTCGTTAG |
| ENSMUSG00000037321 | *Tap1* | TTCAGGATAAGACAGTTC | ATGGTGATGTTGTAGATT |
| ENSMUSG00000045322 | *Tlr9* | ATGGACAGGTGTAAGAAC | ACATCTCTGGCTTGATAG |
| ENSMUSG00000028599 | *Tnfrsf1b* | TCCAAGCATCCTTACATC | TCCAACAATCAGACCAAT |

*Reference genes

Table S5 Statistic analyses of the normalized expression levels of selected 36 genes in the mouse cohort.

| Gene name | Mice No. (n) | | | *P* value between groups | *P* value of pairwise comparison | | |
| --- | --- | --- | --- | --- | --- | --- | --- |
|  | Group C | Group E | Group M |  | E vs. C | E vs. M | M vs. C |
| *C3* | 51 | 42 | 45 | 0.000 | 0.000 | 0.000 | 1.000 |
| *Ccr7* | 50 | 41 | 49 | 0.000 | 0.000 | 0.000 | 1.000 |
| *Cd19* | 50 | 43 | 48 | 0.000 | 0.000 | 0.000 | 1.000 |
| *Cd27* | 51 | 40 | 50 | 0.001 | 0.000 | 0.067 | 0.324 |
| *Cd4* | 51 | 42 | 48 | 0.000 | 0.000 | 0.000 | 1.000 |
| *Cd40* | 49 | 42 | 42 | 0.000 | 0.000 | 0.000 | 1.000 |
| *Cdkn1a* | 51 | 43 | 49 | 0.000 | 0.000 | 0.023 | 0.051 |
| *Clca3a1* | 10 | 3 | 8 | 0.082 | NA | NA | NA |
| *Clcf1* | 51 | 43 | 49 | 0.000 | 0.000 | 0.000 | 1.000 |
| *Clec4g* | 51 | 41 | 49 | 0.000 | 0.000 | 0.000 | 1.000 |
| *Ctsw* | 50 | 40 | 45 | 0.000 | 0.000 | 0.000 | 1.000 |
| *Cxcr4* | 51 | 43 | 48 | 0.000 | 0.000 | 0.000 | 1.000 |
| *Cxcr5* | 49 | 43 | 49 | 0.000 | 0.000 | 0.000 | 1.000 |
| *F13a1* | 33 | 32 | 37 | 0.159 | NA | NA | NA |
| *Fcgr2b* | 49 | 42 | 50 | 0.000 | 0.000 | 0.006 | 0.008 |
| *Gzmb* | 40 | 39 | 46 | 0.000 | 0.000 | 0.000 | 1.000 |
| *H2-Ab1* | 51 | 43 | 48 | 0.000 | 0.000 | 0.000 | 1.000 |
| *H2-Q7* | 50 | 42 | 48 | 0.000 | 0.000 | 0.000 | 0.137 |
| *Icam1* | 37 | 37 | 37 | 0.000 | 0.000 | 0.000 | 1.000 |
| *Icosl* | 50 | 43 | 49 | 0.000 | 0.000 | 0.000 | 1.000 |
| *Ifng* | 6 | 2 | 10 | 0.271 | NA | NA | NA |
| *Il10ra* | 51 | 42 | 47 | 0.000 | 0.000 | 0.000 | 1.000 |
| *Il2rb* | 51 | 42 | 44 | 0.000 | 0.000 | 0.000 | 1.000 |
| *Il4ra* | 47 | 40 | 50 | 0.000 | 0.000 | 0.000 | 0.940 |
| *Itgb7* | 51 | 44 | 48 | 0.000 | 0.000 | 0.000 | 1.000 |
| *Klrg1* | 34 | 35 | 31 | 0.000 | 0.001 | 0.000 | 0.733 |
| *Kmt2d* | 50 | 43 | 50 | 0.002 | 0.002 | 0.698 | 0.060 |
| *Ldlr* | 49 | 38 | 47 | 0.000 | 0.000 | 0.000 | 1.000 |
| *Ly6e* | 51 | 43 | 47 | 0.000 | 0.000 | 0.000 | 1.000 |
| *Prf1* | 5 | 2 | 4 | 0.186 | NA | NA | NA |
| *S100a9* | 50 | 43 | 47 | 0.328 | NA | NA | NA |
| *Siglecg* | 51 | 44 | 49 | 0.000 | 0.000 | 0.001 | 0.497 |
| *Spn* | 50 | 43 | 49 | 0.000 | 0.000 | 0.000 | 1.000 |
| *Tap1* | 51 | 42 | 47 | 0.000 | 0.000 | 0.000 | 1.000 |
| *Tlr9* | 51 | 42 | 49 | 0.000 | 0.000 | 0.000 | 1.000 |
| *Tnfrsf1b* | 51 | 41 | 46 | 0.000 | 0.000 | 0.000 | 1.000 |

*P* value between groups, Kruskal-Wallis non-parametric test.

*P* value of pairwise comparison, adjusted *P* value of Kruskal-Wallis non-parametric test for 3 groups.

NA, not applicable for pairwise comparisons.

Group C/E/M, number of samples with valid Ct values out of the total number of samples in each group. (C: negative control (total *n* = 51); E: early-early tumor (total *n* = 44); M: macroscopic melanoma (total *n* = 50)).

Table S6 Coefficients derived from ridge regression

| E vs. C | |  | E vs. M | |
| --- | --- | --- | --- | --- |
| Variable | coefficient |  | Variable | coefficient |
| *C3* | 0.060 |  | *C3* | 0.084 |
| *Ccr7* | 0.015 |  | *Ccr7* | -0.275 |
| *Cd19* | -0.195 |  | *Cd19* | -0.153 |
| *Cd27* | 0.075 |  | *Cd27* | 0.120 |
| *Cd4* | -0.063 |  | *Cd4* | -0.378 |
| *Cd40* | 0.020 |  | *Cd40* | 0.070 |
| *Cdkn1a* | -0.206 |  | *Cdkn1a* | 0.152 |
| *Clcf1* | 0.039 |  | *Clcf1* | -0.058 |
| *Clec4g* | 0.136 |  | *Clec4g* | 0.003 |
| *Ctsw* | 0.135 |  | *Ctsw* | 0.154 |
| *Cxcr4* | -0.068 |  | *Cxcr4* | -0.093 |
| *Cxcr5* | -0.138 |  | *Cxcr5* | -0.111 |
| *Fcgr2b* | -0.099 |  | *Fcgr2b* | 0.132 |
| *H2Ab1* | -0.024 |  | *H2Ab1* | 0.059 |
| *H2Q7* | -0.140 |  | *H2Q7* | -0.066 |
| *Icosl* | 0.008 |  | *Icosl* | -0.054 |
| *Il10ra* | -0.027 |  | *Il10ra* | -0.088 |
| *Il2rb* | -0.055 |  | *Il2rb* | -0.093 |
| *Il4ra* | -0.121 |  | *Il4ra* | -0.100 |
| *Itgb7* | -0.021 |  | *Itgb7* | -0.060 |
| *Kmt2d* | -0.150 |  | *Kmt2d* | 0.288 |
| *Ldlr* | 0.045 |  | *Ldlr* | 0.098 |
| *Ly6e* | -0.059 |  | *Ly6e* | -0.250 |
| *S100a9* | 0.198 |  | *S100a9* | -0.070 |
| *Siglecg* | -0.083 |  | *Siglecg* | -0.119 |
| *Spn* | -0.017 |  | *Spn* | -0.012 |
| *Tap1* | -0.230 |  | *Tap1* | -0.172 |
| *Tlr9* | -0.024 |  | *Tlr9* | -0.166 |
| *Tnfrsf1b* | 0.087 |  | *Tnfrsf1b* | 0.113 |
| Intercept | 3.891 |  | *Gzmb* | -0.051 |
|  |  |  | Intercept | 4.180 |

Table S7 Coefficients derived from elastic net regression

| E vs. C | |  | E vs. M | |
| --- | --- | --- | --- | --- |
| Variable | coefficient |  | Variable | coefficient |
| Cd19 | -0.058 |  | Ccr7 | -0.048 |
| Cdkn1a | -0.036 |  | Cd4 | -0.127 |
| Clec4g | 0.006 |  | Cdkn1a | 0.032 |
| Cxcr5 | -0.002 |  | Fcgr2b | 0.010 |
| Fcgr2b | -0.003 |  | Kmt2d | 0.061 |
| Kmt2d | -0.028 |  | Ly6e | -0.105 |
| S100a9 | 0.044 |  | Tnfrsf1b | 0.016 |
| Tap1 | -0.147 |  | Intercept | 1.030 |
| Tnfrsf1b | 0.062 |  |  |  |
| Intercept | 1.328 |  |  |  |

Table S8 Root mean square error (RMSE) analyses of ridge, lasso and elastic net regression

|  | Min. | 1st Qu. | Median | Mean | 3rd Qu. | Max. | NA's |
| --- | --- | --- | --- | --- | --- | --- | --- |
| ridge (E vs. C) | 0.221932 | 0.289559 | 0.391673 | 0.363995 | 0.429896 | 0.477914 | 0 |
| lasso (E vs. C) | 0.178717 | 0.273874 | 0.360461 | 0.3428 | 0.408698 | 0.460648 | 0 |
| elastic (E vs. C) | 0.195946 | 0.282426 | 0.358486 | 0.349443 | 0.419391 | 0.466394 | 0 |
| ridge (E vs. M) | 0.19562 | 0.311681 | 0.36604 | 0.356369 | 0.408548 | 0.5115 | 0 |
| lasso (E vs. M) | 0.191831 | 0.281614 | 0.37533 | 0.34606 | 0.388472 | 0.537525 | 0 |
| elastic (E vs. M) | 0.181551 | 0.280964 | 0.376272 | 0.345688 | 0.39035 | 0.537279 | 0 |
